# Metabolic signatures and diagnostic models of ischemic stroke and its hypertensive subtype: a non-targeted metabolomics and machine learning

**DOI:** 10.3389/fgene.2026.1916487

**Published:** 2026-09-22

**Authors:** Xiangjun Kong, Liangying Zhang, Xingwang Li, Qiqi Zhou

**Affiliations:** 1 Central Laboratory, Cangzhou Central Hospital, Cangzhou, China; 2 Scientific Research Office, Cangzhou Central Hospital, Cangzhou, China

**Keywords:** biomarkers, diagnostic model, hypertension, ischemic stroke, machine learning, metabolomics

## Abstract

**Background:**

Ischemic stroke (IS) is a leading cause of death and disability worldwide, yet reliable early diagnostic biomarkers remain lacking. This study employed non-targeted metabolomics and machine learning to characterize metabolic profiles and identify diagnostic biomarkers for IS.

**Methods:**

Non-targeted metabolomics profiling was performed using LC-MS/MS on plasma samples from 100 IS patients, including 50 cases of hypertensive ischemic stroke (HIS) and 50 cases of non-hypertensive ischemic stroke (NHIS), together with metabolomics data of 30 healthy controls (HC) from a previously published studies. Three machine learning algorithms, LASSO, glmBoost, and plsRglm, were used to screen candidate metabolite markers and construct a diagnostic model. Furthermore, targeted metabolomics was performed for quantitative validation of core differential metabolites in an independent clinical cohort.

**Results:**

A total of 59 significantly differential expressed metabolites (DEMs) between IS and HC were identified, mainly enriched in Ferroptosis, Glutathione metabolism, and Sphingolipid signaling pathways. L-Cystine, LPC 18:1, and LPC 14:0 were selected as candidate markers. The diagnostic model constructed based on these three metabolites achieved area under the curve (AUC) values of 0.901 (95% CI: 0.852–0.950) and 0.860 (95% CI: 0.798–0.879) in the training and validation sets, respectively. Targeted metabolomics validated the expression trends of these three metabolites, consistent with the untargeted findings. 50 DEMs were identified between HIS and NHIS, mainly involving amino acid biosynthesis, ether lipid metabolism, and glycerophospholipid metabolism. Indole metabolites were synergistically upregulated, and serum TNF-α levels were significantly increased in the HIS (*P* < 0.05). A total of 58 DEMs were identified between HIS and HC, mainly enriched in sphingolipid signaling pathway, ferroptosis, glutathione metabolism, and nucleotide metabolism. The diagnostic model constructed based on three metabolites screened by machine learning achieved AUCs of 0.883 (95% CI: 0.833–0.983) and 0.790 (95% CI: 0.712–0.816) in the training and validation set, respectively.

**Conclusion:**

This study systematically delineated the metabolic reprogramming characteristics of IS, constructed diagnostic models for IS and HIS stroke with good diagnostic performance, and preliminarily validated the reliability of L-Cystine, LPC 18:1, and LPC 14:0 as candidate diagnostic markers, providing a scientific basis for the early diagnosis and precise intervention of IS.

## Introduction

IS is a common cerebrovascular disease that frequently occurs in middle-aged and elderly individuals. It ranks as the second leading cause of death and a major cause of disability worldwide (Virani et al., 2021; GBD, 2021 Stroke Risk Factor Collaborators, 2024). As a multifactorial and heterogeneous neurological disorder, IS is associated with high mortality and long-term impairment (Cui and Naikoo, 2019). The timing of diagnosis and intervention plays a crucial role in patients’ prognosis and quality of life. However, reliable biomarkers for early diagnosis and prognostic prediction of IS remain scarce, limiting accurate identification and intervention at an early stage (Liang et al., 2025). Currently, the clinical diagnosis of stroke primarily relies on neuroimaging examinations such as computed tomography (CT) and magnetic resonance imaging (MRI). Although these methods can effectively identify the location and extent of infarction, their application still has limitations in ultra-early stages, tiny lesions, or resource-limited areas (Oppenheim et al., 2000). Therefore, there is an urgent need for reliable biomarkers for early diagnosis. Hypertension is not only the most critical modifiable risk factor for stroke, with approximately 70%–75% of stroke patients having concurrent hypertension, but it also complicates their metabolic and pathological mechanisms (George et al., 2017; Webb and Werring, 2022), suggesting that the HIS may require specific diagnostic strategies. Currently, inflammatory and thrombosis-related biomarkers such as D-dimer, lipoprotein(a), and high-sensitivity C-reactive protein have been explored in the field of stroke, but their clinical value remains to be improved due to insufficient sensitivity and specificity (Barakzie et al., 2023; Luo et al., 2025; Rosenberg et al., 2020; Schutte et al., 2016). To address these challenges, this study employed non-targeted metabolomics combined with machine learning to systematically characterize the metabolic profiles of ISand HIS, and to construct diagnostic models for early detection and precise risk stratification.

Metabolomics is an important component of systems biology, serving as a comprehensive analytical approach for small-molecule metabolites produced by cells, tissues, or organisms during a specific period (Michely and Maurer, 2018). In recent years, metabolomics has shown great potential in biomarker discovery and is increasingly used in research areas such as disease diagnosis, drug target identification, mechanism elucidation, and treatment outcome monitoring (Monteiro et al., 2013; Sethi and Brietzke, 2015). In recent years, machine learning methods have demonstrated broad application prospects in disease diagnosis and biomarker screening, owing to their powerful modeling capabilities for high-dimensional complex data. In pancreatic ductal adenocarcinoma, multi-omics analysis combined with machine learning methods has successfully identified a biomarker panel composed of 21 proteins, 4 lipids, and 13 metabolites, providing a new candidate tool for early detection (Devasahayam Arokia Balaya et al., 2025). In breast cancer research, a 4-metabolite diagnostic model containing inosine and uridine was constructed based on metabolomic analysis, demonstrating good early detection performance in an independent validation cohort (Song et al., 2024). In studies on coronary artery disease, triglycerides and apolipoprotein B within low-density lipoprotein have been found to be significantly associated with disease severity, and metabolomic indices can predict the risk of major adverse cardiovascular events (Mei et al., 2024). The above progress provides robust methodological support for the application of metabolomics in the study of diagnostic biomarkers for ischemic stroke. In the field of ischemic stroke, some studies have already attempted to use metabolomics to screen for diagnostic biomarkers. Zhao et al., based on serum samples from 44 IS patients and 44 healthy controls, conducted a combined analysis of untargeted metabolomics and proteomics to identify differential metabolites and construct a diagnostic model (Zhao et al., 2024). Li et al. found that the combination of biliverdin and nicotinamide N-oxide can accurately identify acute IS patients within 4.5 h of symptom onset (Li et al., 2025). Although the above studies have demonstrated the potential of metabolomics in the diagnosis of IS, the field as a whole is still in an exploratory stage and requires further systematic research.

In this study, we performed non-targeted metabolomics analysis on plasma samples from patients with IS, systematically characterizing the metabolic profiles of stroke and its hypertensive subgroup. Subsequently, machine learning methods were used to construct diagnostic models and screen for key metabolites with potential diagnostic value. Finally, targeted metabolomics technology was applied for quantitative validation of core differential metabolites in an independent clinical cohort. This study aims to explore serum metabolic markers that can be used for early diagnosis of IS and risk stratification of HIS, providing scientific evidence for early diagnosis and personalized treatment.

## Materials and methods

### Study subjects and data collection

A total of 130 subjects were enrolled for metabolomic analysis in the present study, consisting of three independent groups: 50 patients with HIS, 50 patients with NHIS, and 30 HC. The 100 IS patients (50 HIS and 50 NHIS) were prospectively recruited at Cangzhou Central Hospital. Venous blood samples were collected from all patients. The baseline clinical and demographic characteristics of IS patients are summarized in Supplementary Table S1. Plasma samples were collected before treatment and stored at −80 °C until sample preparation and ultra-high performance liquid chromatography-tandem mass spectrometry (LC-MS/MS) analysis. This study followed the ethical guidelines of the Declaration of Helsinki. All participants fully understood the study purpose, procedures, potential risks and benefits before enrollment, and voluntarily signed written informed consent. The study protocol was approved by the Ethics Committee of Cangzhou Central Hospital (Approval No.: 2024-044-01). The metabolomics data of 30 HC used in this study were derived from previously published studies (Ren et al., 2026). According to the original study, the HC subjects were healthy individuals with normal lung function, absence of respiratory symptoms, and no severe cardiovascular, hepatic, renal, or metabolic diseases, which qualifies them as an appropriate healthy control reference for the present study. The baseline clinical and demographic characteristics of all participants are summarized in Supplementary Table S1. To minimize inter-dataset technical variations, we performed log2 transformation and Z-score normalization within each dataset, followed by batch effect correction using the ComBat method (Johnson et al., 2007) implemented in the sva R package, with dataset source as the batch variable.

### Sample preparation

After thawing the plasma samples on ice, take 50 µL of plasma and add extraction solvent (80% methanol containing 10 µL of mixed isotope internal standard solution) to a final volume of 200 μL, then vortex mix for 20 min for metabolite extraction and protein precipitation. After mixing, centrifuge the samples at 12,000 g and 4 °C for 10 min. After centrifugation, lyophilize the entire supernatant. For analysis, reconstitute the lyophilized sample with 200 µL of reconstitution solvent (0.1% FA), and inject 10 µL for mass spectrometry quantification.

### LC-MS/MS

All samples were analyzed using a Vanquish Ultra-High Performance Liquid Chromatograph (Thermo Scientific, United States) coupled with a Q Exactive quadrupole-Orbitrap high-resolution mass spectrometer (Thermo Scientific, United States). To expand the coverage of metabolites, separation was performed using both reversed-phase chromatography and hydrophilic interaction chromatography, and data were acquired in positive and negative ion modes of electrospray ionization (ESI), respectively. For reversed-phase chromatography separation, an Acquity™BEH C18 column (Waters Co., United States) was used. The mobile phase consisted of water/methanol (both containing 0.1% formic acid and 5 mM ammonium formate), with a gradient elution program as follows: the organic phase was linearly increased from 2% to 100% within 12 min, followed by flushing and equilibrating the column with 100% organic phase for 6 min. The flow rate was 0.3 mL/min, and the column temperature was 40 °C. Hydrophilic interaction chromatography separation was performed using an Acquity™BEH AMIDE column (Waters Co., United States). The mobile phase consisted of water/acetonitrile (both containing 25 mM ammonium formate, pH adjusted to 9.0). The gradient elution program was as follows: the organic phase was linearly increased from 2% to 98% within 10 min, followed by flushing and re-equilibrating the column with 98% organic phase for 2 min. The flow rate was 0.4 mL/min, and the column temperature was 40 °C. Mass spectrometry detection was performed using a heated electrospray ionization source with an ionization voltage of 4 kV in positive ion mode and 3.5 kV in negative ion mode. The sheath gas flow rate was 45 arb, the auxiliary gas flow rate was 10 arb, the heater temperature was 355 °C, the capillary temperature was 320 °C, and the ion transfer tube RF level was 50%. Data-dependent acquisition (DDA) mode was used to collect mass spectrometry data. The full scan resolution was set to 70,000 (@ m/z 200), the automatic gain control (AGC) was 1 × 10^6^, the maximum ion injection time was 100 m, and the scan m/z range was 70–1050. The MS/MS resolution was 17,500 (@ m/z 200), the AGC was 1 × 10^5^, the maximum ion injection time was 50 m, the scan m/z range was 70–1050, the normalized collision energy was 40%, and ultrapure nitrogen was used as the fragmentation gas.

### Quality control (QC) for metabolomics

Metabolites intensities were exported from Compound discoverer 3.2. Metabolites with proportion of missing values greater than 0.2 were discarded, then all missing values were imputated with KNN (K-Nearest Neighbors). When applying the Systematic Error Removal using Random Forest (SERRF) approach for normalization, all sample types were classified as biological samples excluding the “pooled QC samples”. Subsequently, the correction was performed using the ‘QC samples’ present in each batch, fostering accuracy and consisteHPy across the diversified data set. Relative standard deviation (RSD) of metabolites intensities were calculated within the group according to the sample using numpy package (v.1.21.0) in Python. Pearson correlations between samples were calculated using the ‘cor’ fuHPtion and pheatmap package (v.1.012) in R. PriHPipal component analysis (PCA) was applied to with ‘prcomp’ fuHPtion in R (v.4.1.0).

### Metabolite identification and quantification

DDA raw data was extracted, peak features-identified and QC processed using Compound Discoverer v 3.2 software. Peaks were quantified using area-under-thecurve. Compounds were identified by comparison to library entries of purified standards or public MS/MS entities. Q-life lab maintains a library based on authenticated standards that contains the retention time (RT), mass to charge ratio (m/z), and chromatographic data (iHPluding MS/MS spectral data) on all molecules present in the library with mzValt 2.3. Other public libraries such as NIST 2022, mzCloud 2021B and mzCloud 2023 were used for metabolites identification. Furthermore, 32 biochemical identifications as level 1 are based on three criteria: RT within a narrow window of the proposed identification, accurate mass match to the library ± 10ppm, and the mzValt scores between the experimental data and authentic standards. The MS/MS scores are based on a comparison of the ions present in the experimental spectrum to the ions present in the library spectrum, filtered greater than 0.6. While there may be similarities between these molecules based on one of these factors, the use of all three data points can be utilized to distinguish and differentiate biochemicals.

### Metabolite data analysis

After missing value imputation, normalization, and log2 transformation, fold changes between comparison groups were calculated. DEMs were identified using two-sided Welch’s unpaired t-test implemented in the stats package of R software (version 4.4.0), with *P* < 0.05 considered statistically significant. Kyoto encyclopedia of genes and genomes (KEGG) pathway enrichment analysis was performed using the MetaboAnalyst 6.0 platform (https://www.metaboanalyst.ca/). Hypergeometric test was used for over-representation analysis, with *P* < 0.05 as the threshold for significantly enriched pathways. Mfuzz clustering analysis was performed using the Mfuzz R package. Briefly, the metabolite abundance matrix was first Z-score transformed. The fuzzification parameter m was estimated using the mestimate function. Metabolites were assigned to clusters based on their membership coefficients, resulting in two distinct expression patterns (relatively decreased or relatively increased) in HIS. This analysis was used to visualize overall expression trends of differential metabolites and was not intended as an additional criterion for DEM screening.

### Diagnostic model construction

LASSO regression was implemented using the glmnet package (v4.1–8) in R software (version 4.4.0), with disease status as the binary outcome variable (family = “binomial”, alpha = 1). The optimal penalty parameter λ was determined via 10-fold cross-validation, and metabolites with non-zero coefficients at lambda.1se were selected to obtain a relatively parsimonious feature set. glmBoost analysis was performed using the mboost package (v2.9–11) with a binomial response model, and the optimal number of boosting iterations (mstop) was determined via cross-validation. Partial least squares generalized linear models were constructed using the plsRglm package (v1.6.0), with the optimal number of latent variables selected based on cross-validation results. For the comparison between IS and HC, only metabolites commonly identified by all three algorithms (LASSO, glmBoost, and plsRglm) were retained. For the comparison between HIS and HC, only metabolites commonly identified by LASSO and glmBoost were retained. The selected core metabolites were then incorporated into binary logistic regression models for diagnostic model construction. Samples were stratified by disease status and randomly divided into training and internal hold-out validation sets at a 7:3 ratio. Receiver operating characteristic (ROC) curves were plotted using the pROC package (v1.18.5) to calculate the AUC, 95% confidence intervals, sensitivity, and specificity. The optimal diagnostic cut-off value was determined based on the Youden index. This validation was performed as an internal hold-out validation and should not be interpreted as external validation. All statistical analyses were performed using R software (version 4.4.0).

### Targeted metabolomics validation of key metabolites

To validate the key metabolites identified by previous untargeted metabolomics, this study employed targeted metabolomics for quantitative validation in an independent clinical cohort. This validation cohort, independent of the initial discovery cohort, included 6 IS and 6 HC. All participants were fully informed of the study purpose, procedures, and potential risks before enrollment and voluntarily signed written informed consent forms. The study protocol was approved by the Ethics Committee of Cangzhou Central Hospital (Approval No. 2024-044-01). Peripheral venous blood was collected from all subjects, plasma was separated and stored at −80 °C until analysis. Targeted quantitative analysis of target metabolites in plasma samples was performed using LC-MS/MS. Calibration curves were established using standards to calculate the concentrations of metabolites in plasma. Orthogonal partial least squares discriminant analysis (OPLS-DA) was used to evaluate the overall metabolic profile differences between the two groups. The Wilcoxon rank-sum test was employed to compare the differences in metabolite expression levels between the stroke group and the healthy control group, with *P* < 0.05 considered the threshold for statistical significance.

## Results

### Metabolic profiling characteristics and establishment of a diagnostic model in IS

After performing PCA on the metabolomic data of 100 IS and HC, the results showed that the two groups exhibited clear separation in principal components (Figure 1A), indicating systematic metabolic differences between the two groups. Using a threshold of fold change ≥1.5 and *P* < 0.05, DEMs between the two groups were screened. A total of 59 DEMs were identified, of which 34 were upregulated and 25 were downregulated in IS compared to HC. The volcano plot visually displays the distribution of these DEMs (Figure 1B). Significantly upregulated differential metabolites include L-glutamic acid, bilirubin, sphingosine-1-phosphate. KEGG pathway enrichment analysis of the DEMs showed that they were significantly enriched in Ferroptosis, Glutathione metabolism, and Sphingolipid signaling pathway (Figure 1C). These results indicate that patients with IS exhibit significant oxidative stress imbalance and sphingolipid metabolism disorder, and changes in these metabolic pathways may collectively participate in the pathogenesis of ischemic stroke. To improve the robustness of biomarker screening, three algorithms—LASSO, glmBoost, and plsRglm—were respectively used for variable selection, and the intersection of the three was taken as the final result. A total of three candidate metabolite biomarkers, L-Cystine, LPC 18:1, and LPC 14:0, were obtained (Figure 1D). Subsequently, each group of samples was randomly divided into a training set and a validation set at a ratio of 70%–30%, and a diagnostic model was constructed based on the three selected metabolites. The results showed that the AUC of the model in the training set and validation set were 0.901 (95% CI: 0.852–0.95) and 0.860 (95% CI: 0.798–0.879), respectively (Figures 1E,F), indicating its good diagnostic performance.

**FIGURE 1:**
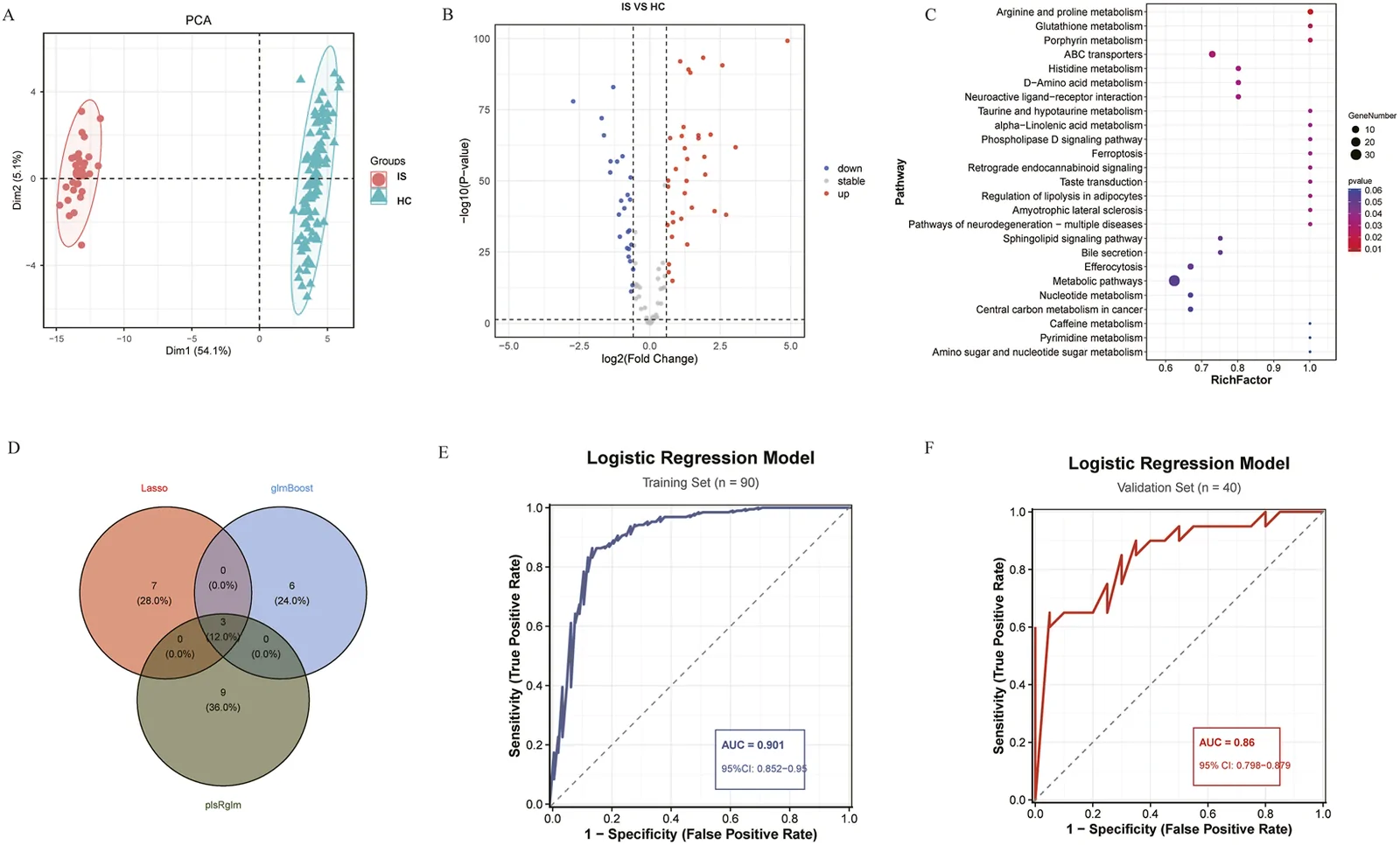
Differential metabolic profiles between IS and HC and the diagnostic performance of candidate biomarkers. **(A)** PCA analysis of metabolites in the two groups. **(B)** Volcano plot of significantly different metabolites between the two groups. **(C)** KEGG pathway enrichment analysis of DEMs between the two groups. **(D)** Venn diagram of candidate biomarkers screened by three algorithms. **(E,F)** ROC curves of the diagnostic model constructed based on three candidate metabolites in the training set **(E)** and validation set **(F)**.

### Comparative analysis of metabolic profiles between HIS and NHIS

To investigate the differences in metabolic characteristics between IS and NHIS, this study performed metabolomics analysis on two groups of samples. The results showed that a total of 50 DEMs were identified, among which 27 metabolites were upregulated and 23 metabolites were downregulated in HIS patients compared to NHIS (*P* < 0.05) (Figure 2A). The volcano plot displayed the distribution of these differential metabolites (Figure 2B). The distribution of the types of significantly differential metabolites is shown in Figure 2C, mainly including carboxylic acids and their derivatives, glycerophospholipids, indoles and their derivatives, organic sulfuric acids and their derivatives, steroids and their derivatives, benzene and its substituted derivatives, benzopyrans, glycerides, and hydroxy acids and their derivatives. To further reveal the biological functions involved in these differential metabolites, KEGG pathway enrichment analysis was performed. The results showed that the significantly differential metabolites were mainly enriched in Biosynthesis of amino acids, Ether lipid metabolism, and glycerophospholipid metabolism (Figure 2D).

**FIGURE 2:**
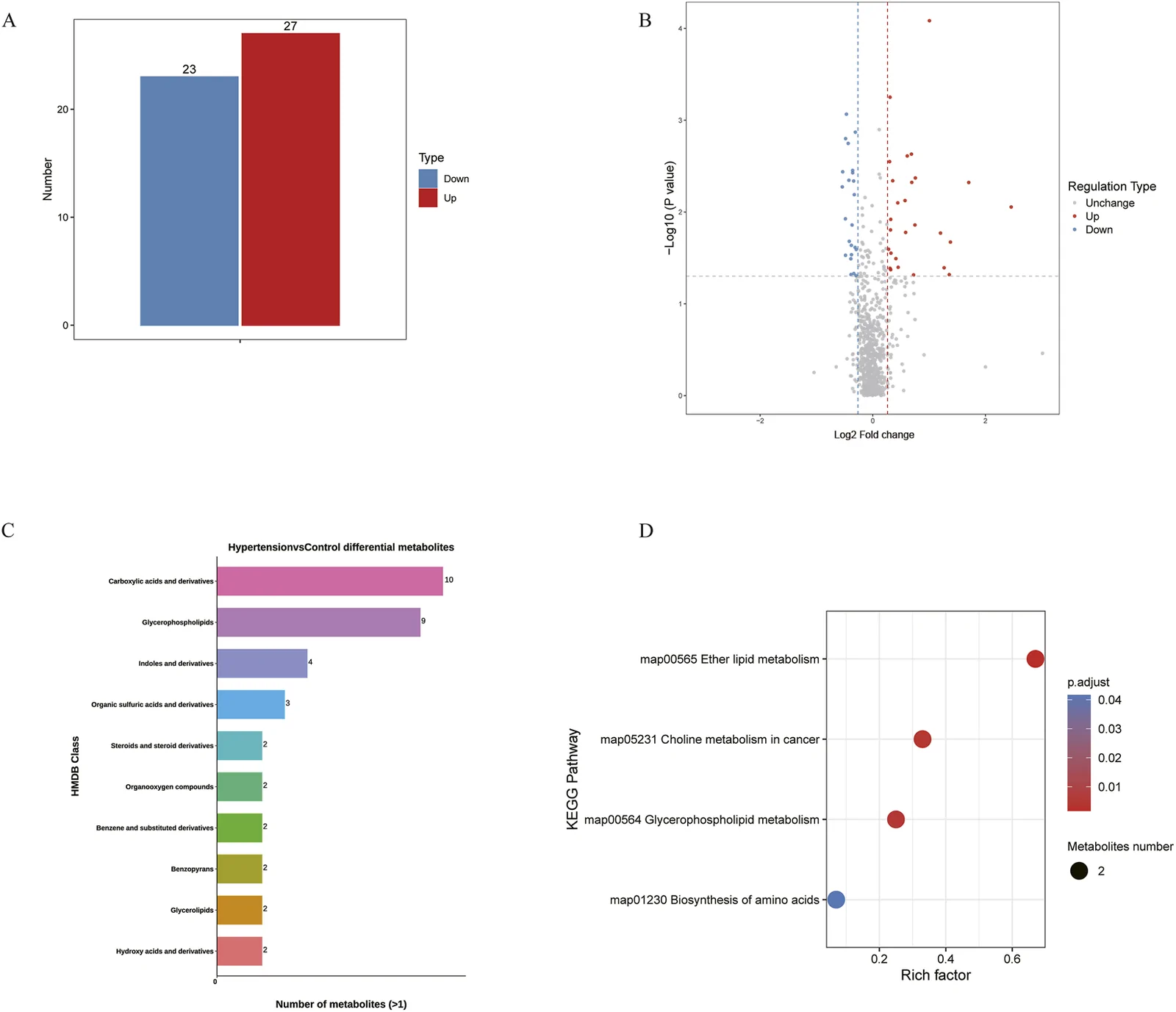
Analysis of metabolic profile differences between HIS and NHIS. **(A)** Statistics of the number of DEMs between the two groups, with red and blue representing upregulated and downregulated metabolites, respectively. **(B)** Volcano plot of DEMs between the two groups. **(C)** Classification statistics of significantly differential metabolites. **(D)** KEGG pathway enrichment analysis of DEMs.

Mfuzz clustering analysis divided the significantly differential metabolites into two clusters with distinct expression trends (Figure 3A). Metabolites in Cluster 1 showed significantly decreased expression levels in patients with HIS, while metabolites in Cluster 2 exhibited significantly increased expression trends. Metabolites significantly downregulated in the hypertension stroke group mainly include lysophosphatidylcholines (such as LysoPC(O−18:0/0:0), LysoPC(18:1 (9Z)/0:0), LysoPC(18:2 (9Z,12Z)/0:0), LysoPC(24:0/0:0), and LysoPC(P−18:0/0:0)) and glycerophosphorylcholine (Figure 3B). In the HIS group, Indoxyl sulfate, N-Acetyltryptophan, and Tauroursodeoxycholic acid (TUDCA) were significantly higher than in the NHIS (Figure 3C). To further explore the interactions among these differential metabolites, a correlation analysis was performed on them. The results showed a complex correlation network among the metabolites (Figure 3D). Among the indole metabolites closely associated with HIS, including 1H-Indol-2-ol, 5,6-Dihydroxyindole, 5-Hydroxyindole, and Indoxyl sulfate, a significant positive correlation was observed, suggesting that these tryptophan-derived metabolites may collaboratively participate in oxidative stress and inflammatory responses. Given the potential association between the aforementioned indole metabolites and inflammation, we further measured serum levels of TNF-α in two groups. The results showed that, compared with the NHIS, TNF-α was significantly elevated in the HIS (*P* < 0.05) (Figure 3E).

**FIGURE 3:**
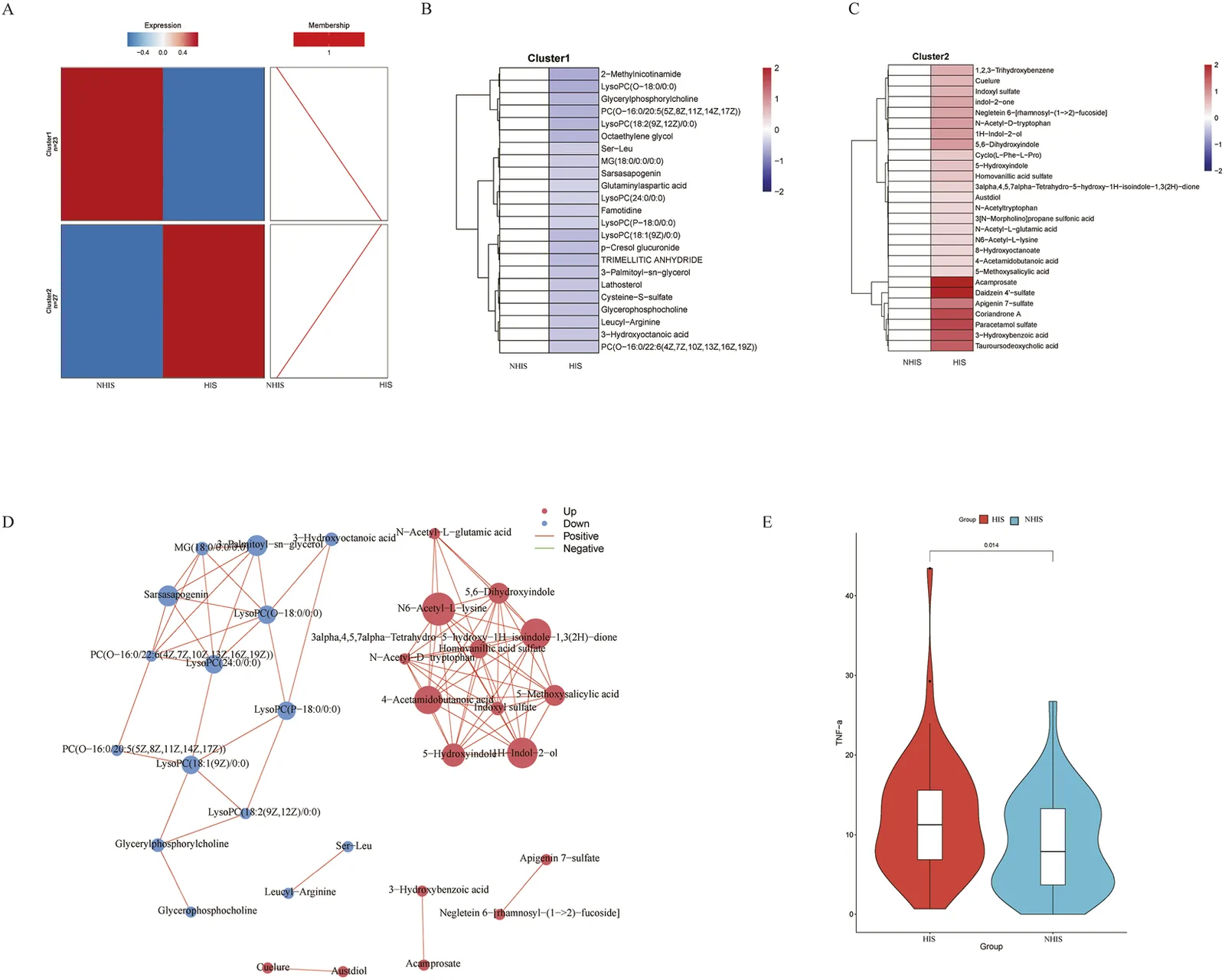
Cluster analysis, correlation analysis, and inflammatory factor analysis of differential metabolites. **(A–C)** Mfuzz clustering analysis divided the DEMs into two clusters **(A)**. Cluster 1 represents metabolites with decreased expression in the HIS **(B)**, and cluster 2 represents metabolites with increased expression in HIS **(C)**. **(D)** Correlation heatmap of differential metabolites between the two groups. **(E)** Comparison of TNF-α expression levels between the two groups.

### Metabolomic analysis and diagnostic model construction in HIS

The above results indicate that patients with IS exhibit significant metabolic disorder characteristics, and hypertension, as an important risk factor for stroke, may further exacerbate metabolic abnormalities. To explore the specific metabolic features and diagnostic value of HIS, this study further conducted a comparative metabolomics analysis between HIS and HC. PCA results showed a clear separation trend between the two groups in the principal component space (Figure 4A), indicating systemic metabolic differences between HIS and HC. Further using the threshold of fold change ≥1.5 and *P* < 0.05 for differential metabolite screening, a total of 58 DEMs were identified, among which 24 metabolites were upregulated and 34 metabolites were downregulated in HIS compared to HC (Figure 4B). KEGG enrichment analysis of DEMs revealed significant enrichment in the Sphingolipid signaling pathway, Ferroptosis, Glutathione metabolism, and Nucleotide metabolism (Figure 4C). To further construct a diagnostic model for distinguishing HIS, two machine learning algorithms were applied to screen the DEMs, and three metabolites were ultimately identified as candidate biomarkers (Figure 4D). A diagnostic model was constructed based on metabolites. The ROC curve analysis showed that the AUC of the model in the training set and validation set were 0.883 (95% CI 0.833–0.983) and 0.79 (95% CI: 0.712–0.816), respectively, indicating good diagnostic performance (Figure 4E).

**FIGURE 4:**
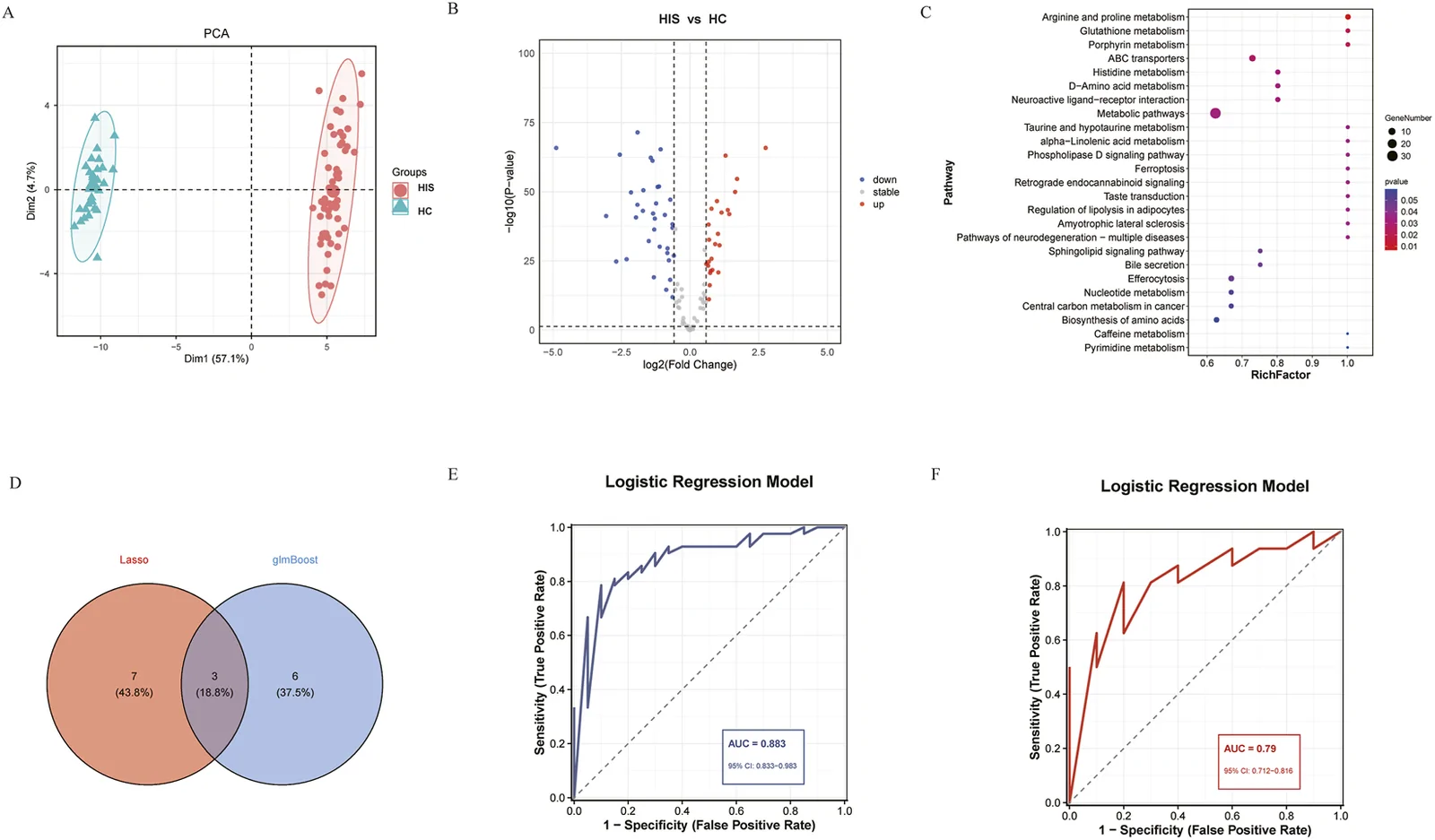
Differences in metabolic profiles between HIS and HC and construction of a diagnostic model. **(A)** PCA of metabolites in two group **(B)** Volcano plot of DEMs between the two groups. **(C)** KEGG pathway enrichment analysis of DEMs. **(D)** Venn diagram of candidate biomarkers screened by two machine learning algorithms, with three metabolites identified in the intersection. **(E,F)** ROC curves of the diagnostic model based on the three candidate metabolites in the training set **(E)** and validation set **(F)**.

### Targeted metabolomics validation of key metabolites

To validate the key DEMs identified through metabolomics screening, this study employed targeted metabolomics for quantitative analysis. In the previous untargeted metabolomics analysis, based on data from 100 IS and 30 HC, three core key metabolites were identified: LysoPC(14:0) was significantly downregulated in IS, while LysoPC (18:1) and L-Cystine were significantly upregulated in IS (Figure 5A). To further verify these findings, targeted metabolomics detection was performed in an independent clinical cohort in this study. This validation cohort included 6 IS patients and 6 HC. Targeted quantitative analysis of serum samples was performed using LC-MS/MS. OPLS-DA results showed a clear trend of cluster separation between the HC and the IS on the score plot, indicating significant differences in the overall metabolic profiles between the two groups (Supplementary Figure S1). Quantitative analysis of these three metabolites showed that in the validation cohort, the expression level of LysoPC (14:0) was decreased in the IS, while the expression levels of LysoPC (18:1) and L-Cystine were increased, consistent with the findings of our results (Figure 5B). The above targeted metabolomics validation results further confirmed the differential expression patterns of LysoPC (14:0), LysoPC (18:1), and L-Cystine in the serum of IS, suggesting that these three metabolites have reliability as potential biomarkers for IS.

**FIGURE 5:**
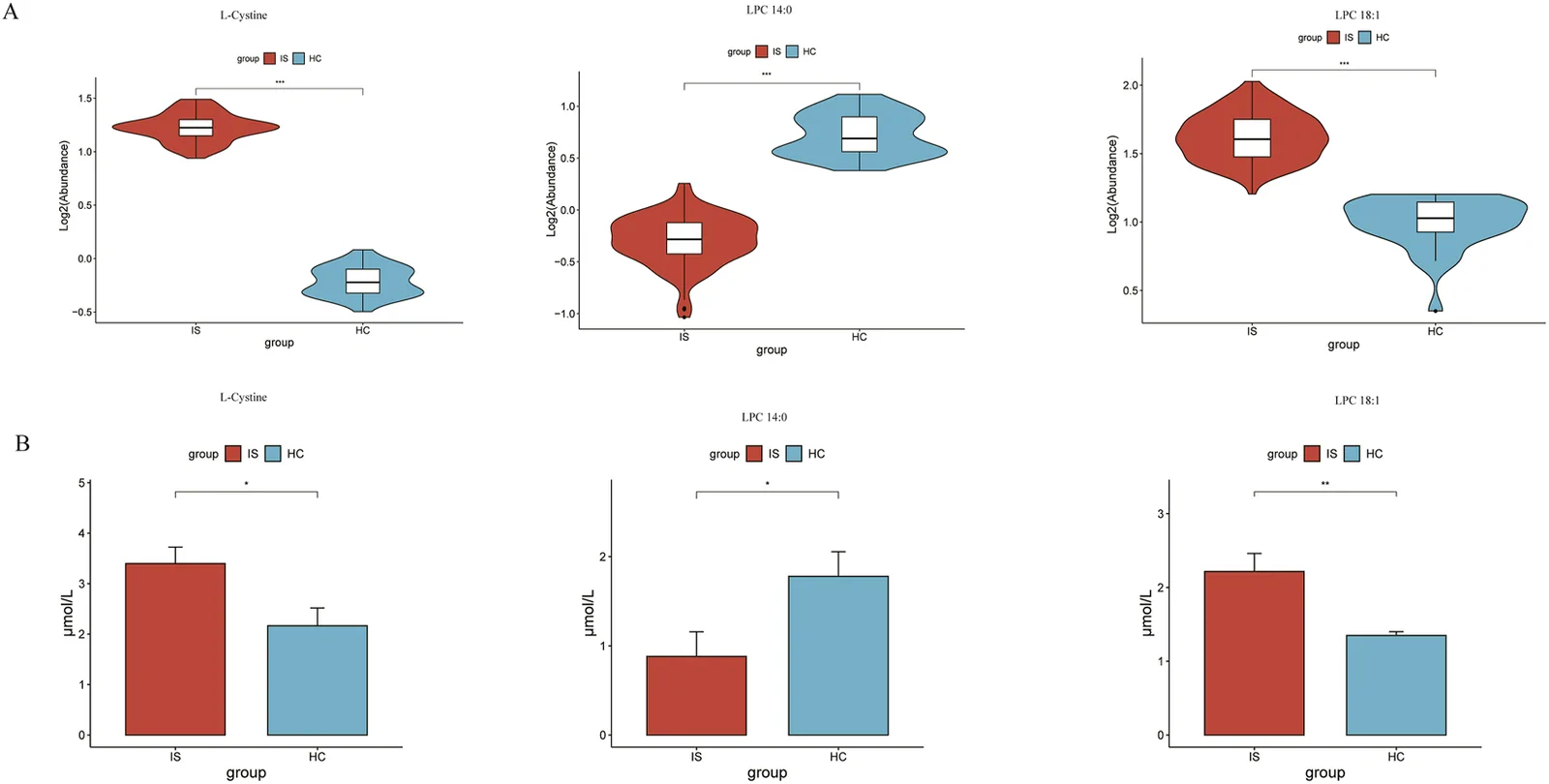
Targeted metabolomics validation of key differential metabolites. **(A)** Comparison of the expression levels of three Key metabolites between the two groups based on non-targeted metabolomics data. **(B)** Validation of the expression analysis of the three metabolites in an independent validation cohort.

## Discussion

IS accounts for 60%–80% of all strokes and is the second leading cause of death and major cause of disability worldwide (Feigin et al., 2025). Despite significant progress in acute-phase treatment, the lack of reliable early diagnostic biomarkers still limits precise intervention (Sun and Wang, 2023). Metabolomics can holistically reflect disease-related metabolic disturbances and offers unique advantages in biomarker discovery. This study systematically analyzed the plasma metabolic profiles of IS using metabolomics technology and constructed a diagnostic model combined with machine learning to explore potential metabolic biomarkers for early diagnosis of IS.

A total of 59 DEMs between IS patients and healthy controls were identified in this study, mainly enriched in ferroptosis, glutathione metabolism, and sphingolipid signaling pathways. Ferroptosis is an important mode of neuronal death in cerebral ischemia-reperfusion injury, and glutathione depletion and GPX4 inactivation after ischemia can lead to enhanced lipid peroxidation (Fei and Leng, 2026). Multi-omics studies have confirmed that the cystine transporter SLC3A2 is significantly downregulated in ischemic brain tissue, the levels of glutathione synthesis precursors such as glutamine and glycine are decreased, and GPX4 expression is reduced, indicating that glutathione metabolic imbalance is a key link in the ferroptosis pathway (Zhang et al., 2023; He et al., 2025). Regarding the sphingolipid signaling pathway, sphingolipid metabolism is one of the main enriched biological processes in the serum of ischemic stroke model mice, and S1P levels are elevated in brain tissue (Liu et al., 2024).

Clinical studies have also confirmed significant alterations in serum levels of sphingolipid metabolites, such as ceramides and sphingosylphosphorylcholine, in patients with acute IS (Yuan et al., 2023; Mohamud Yusuf et al., 2024). These findings suggest that the ferroptosis-glutathione metabolism axis and sphingolipid signaling pathways play a core role in the pathogenesis of IS. Based on this, the present study further identified L-Cystine, LPC 18:1, and LPC 14:0 as candidate diagnostic biomarkers for IS. Cystine deficiency can directly induce neuronal ferroptosis by inhibiting the system Xc-GSH-GPX4 pathway, while cystine overload may exert an antioxidant compensatory effect through activation of the NRF2 signaling pathway (Sun et al., 2023; Li et al., 2022). Untargeted lipidomics studies have identified LPC 14:0 as one of the differentially altered lipid metabolites in acute IS (Guo et al., 2022). Another study confirmed that LPC (18:1) is closely associated with post-stroke sensorimotor dysfunction and the severity of brain injury (Turpin et al., 2024). The above findings support the value of L-Cystine, LPC 18:1, and LPC 14:0 as potential biomarkers for the early diagnosis of IS, and also provide experimental evidence for intervention strategies targeting ferroptosis and sphingolipid metabolic pathways.

Further comparing the metabolic profiles of HIS and NHIS, this study reveals unique metabolic reprogramming features driven by hypertension. Notably, glycerophospholipid metabolism differed significantly between the two groups, and abnormal glycerophospholipid metabolism in hypertensive states has been closely linked to vascular endothelial dysfunctio (Sun et al., 2021). The lipid downregulation observed in this study may reflect a metabolically vulnerable state induced by hypertension, exacerbating the metabolic stress load of stroke. In addition, TUDCA was specifically highly expressed in the HIS group. TUDCA has been shown to attenuate neurological deficits and reduce brain injury by inhibiting endoplasmic reticulum stress-mediated neuronal apoptosis via suppression of the PERK/eIF2α/ATF4 signaling pathway in traumatic brain injury (Meng et al., 2024). Tryptophan metabolic has been confirmed to be involved in the pathological processes of atherosclerosis and stroke (Yao et al., 2026). Serum TNF-α levels were significantly elevated in the HIS. As a key pro-inflammatory cytokine, it is closely associated with vascular endothelial injury and stroke risk in hypertension complicated by cerebral infarction (Zhang et al., 2009). In the comparison between HIS and HC, this study identified 58 DEMs, mainly enriched in sphingolipid signaling pathway, ferroptosis, glutathione metabolism, and nucleotide metabolism. Hypertension is the most important modifiable risk factor for stroke. Large-scale epidemiological surveys have shown that the prevalence of hypertension among stroke survivors in China is approximately 81.5%–84% (Tu et al., 2022). Recent studies have further confirmed that key pathways such as glycerophospholipid metabolism, sphingolipid metabolism, and tryptophan metabolism are significantly disturbed in HIS (Guo et al., 2019). At the mechanistic level, angiotensin II can induce ferroptosis in vascular endothelial cells by inhibiting the System Xc-/GPX4 signaling pathway, while inhibition of ferroptosis can ameliorate vascular endothelial dysfunction (Zhou et al., 2024; Zhang et al., 2022). The above literature reports are highly consistent with the enriched pathways in this study, suggesting that hypertension-related metabolic disorders may drive vascular endothelial injury and ultimately lead to stroke through multiple pathways, including disruption of sphingolipid signaling, promotion of ferroptosis, and impairment of glutathione antioxidant defense.

This study has several limitations. First, the diagnostic models constructed in this study are exploratory and internally validated. Although we reduced the complexity of high-dimensional data modeling by using multiple machine learning algorithms (LASSO, glmBoost, and plsRglm) to screen for overlapping candidate metabolites and by including only a small number of core metabolites in the final models, the single random training/validation split cannot completely avoid feature selection bias and model overfitting. Second, the IS samples in this study were recruited from our hospital, while the HC data were obtained from a public database, rather than being collected simultaneously under the same testing platform and experimental conditions, which may introduce potential confounding between sample source and disease status. Although we unified metabolite annotations and performed within-dataset normalization and matrix-level batch effect correction, the lack of access to raw mass spectrometry data prevented us from implementing fully consistent raw data preprocessing across all cohorts, and residual technical heterogeneity across studies may still persist. Therefore, the conclusions of this study need to be validated using independently collected healthy control samples with a standardized protocol. Third, although we identified differences in the metabolic profiles between HIS and NHIS patients and screened out potential candidate biomarkers, the specific molecular regulatory mechanisms require further in-depth investigation. Future studies should validate our core findings in larger prospective cohorts, and elucidate the upstream regulatory networks and downstream functional effects of key differential metabolites in the pathological progression of IS through cellular and animal experiments.

## Conclusion

This study systematically analyzed plasma samples from patients with IS using non-targeted metabolomics technology to characterize the metabolic profiles of IS. Furthermore, diagnostic models for stroke were constructed using machine learning methods, and a specific diagnostic model for HIS was established. These findings provide novel candidate biomarkers for the early diagnosis of IS and risk stratification of HIS, offer important clues for a deeper understanding of the molecular mechanisms underlying post-stroke metabolic reprogramming, and are expected to provide a scientific basis for the precise diagnosis and treatment of IS.

## Data Availability

The original contributions presented in the study are publicly available. This data can be found in the MetaboLights Human Metabolome Database repository with the accession number: MTBLS15660, at https://www.ebi.ac.uk/metabolights/MTBLS15660.

## Supplementary material

SUPPLEMENTARY FIGURE S1 OPLS-DA of IS and HC in the targeted metabolomics validation cohort.

SUPPLEMENTARY TABLE S1 Baseline clinical and demographic characteristics of the study population.
