# Supplementary material for: Metabolic signatures and diagnostic models of ischemic stroke and its hypertensive subtype: a non-targeted metabolomics and machine learning
Source: Front Genet. 2026 Sep 22;17:1916487. doi: 10.3389/fgene.2026.1916487 (PMC13638189; doi:10.3389/fgene.2026.1916487)
Supplement: Supplementary file 1 [file DataSheet1.pdf]

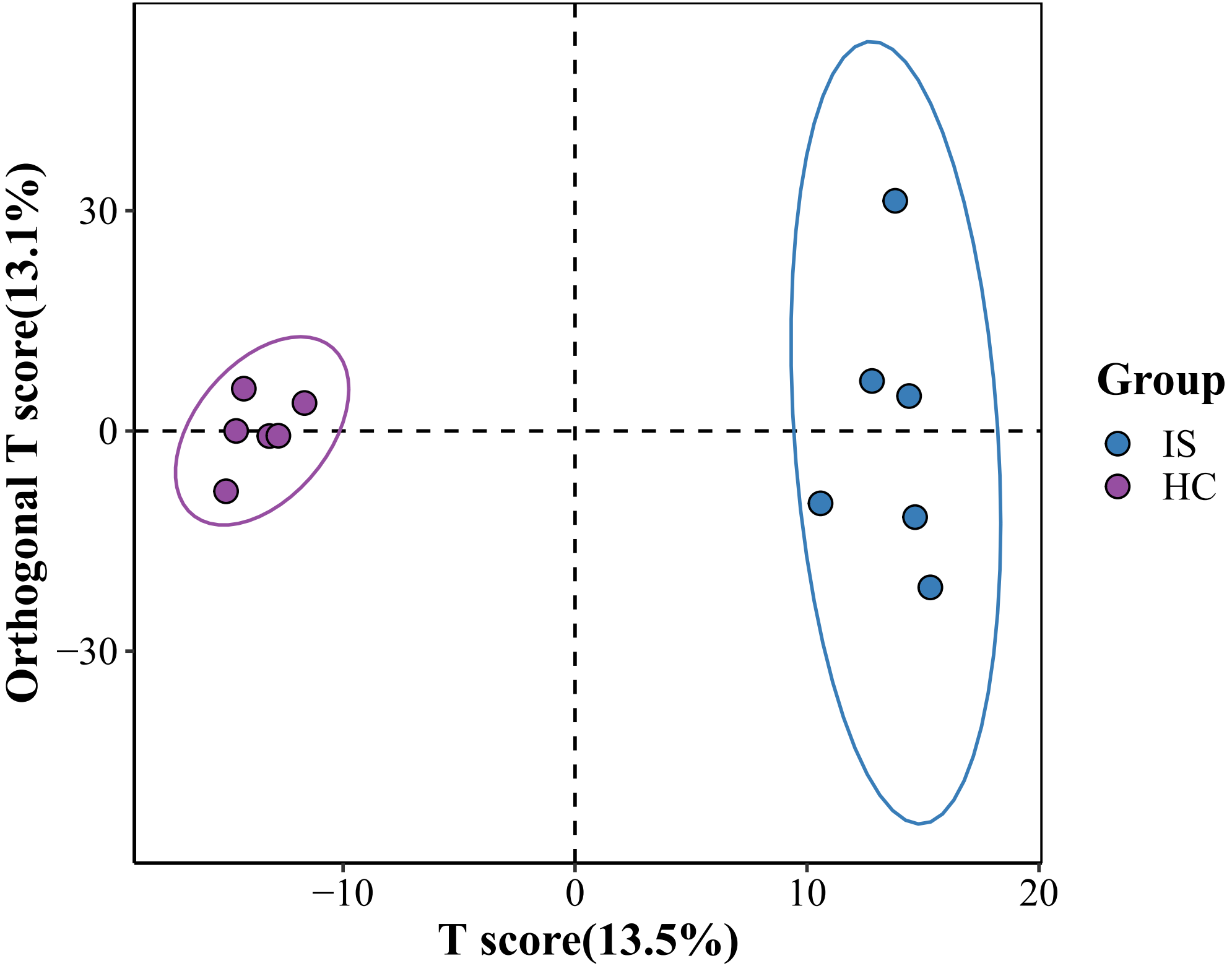

Orthogonal T score(13.1%)
30
0
−30
−10
0
10
20
T score(13.5%)
Group
IS
HC
